# Cell-Based Therapy for the Treatment of Glioblastoma: An Update from Preclinical to Clinical Studies

**DOI:** 10.3390/cells11010116

**Published:** 2021-12-30

**Authors:** Noha Attia, Mohamed Mashal, Sudhakar Pemminati, Adekunle Omole, Carolyn Edmondson, Will Jones, Priyanka Priyadarshini, Temoria Mughal, Pauline Aziz, Blesing Zenick, Ambar Perez, Morgan Lacken

**Affiliations:** 1 The American University of Antigua-College of Medicine, Coolidge 1451, Antigua and Barbuda; PSudhakar@auamed.net (S.P.); aomole@auamed.net (A.O.); cedmondson@auamed.net (C.E.); WillJ@auamed.net (W.J.); PriyankaPr@auamed.net (P.P.); TemoriaM@auamed.net (T.M.); PaulineA@auamed.net (P.A.); BlesingZ@auamed.net (B.Z.); AmbarP@auamed.net (A.P.); MorganL@auamed.net (M.L.); 2 Laboratory of Pharmaceutics, NanoBioCel Group, School of Pharmacy, University of the Basque Country (UPV/EHU), Paseo de la Universidad 7, 01006 Vitoria-Gasteiz, Spain; 3 Histology and Cell Biology Department, Faculty of Medicine, University of Alexandria, Alexandria 21561, Egypt

**Keywords:** glioma, stem cell, CAR-T cell, immune cell therapy, cell therapy, cancer

## Abstract

Glioblastoma (GB), an aggressive primary tumor of the central nervous system, represents about 60% of all adult primary brain tumors. It is notorious for its extremely low (~5%) 5-year survival rate which signals the unsatisfactory results of the standard protocol for GB therapy. This issue has become, over time, the impetus for the discipline of bringing novel therapeutics to the surface and challenging them so they can be improved. The cell-based approach in treating GB found its way to clinical trials thanks to a marvelous number of preclinical studies that probed various types of cells aiming to combat GB and increase the survival rate. In this review, we aimed to summarize and discuss the up-to-date preclinical studies that utilized stem cells or immune cells to treat GB. Likewise, we tried to summarize the most recent clinical trials using both cell categories to treat or prevent recurrence of GB in patients. As with any other therapeutics, cell-based therapy in GB is still hampered by many drawbacks. Therefore, we highlighted several novel techniques, such as the use of biomaterials, scaffolds, nanoparticles, or cells in the 3D context that may depict a promising future when combined with the cell-based approach.

## 1. Introduction

Glioblastoma (GB) is one of the most aggressive and rare malignancies known to be quite difficult to treat. The tumor is subcortical, yet grows rapidly and often invades several deep structures, affecting the contralateral cerebral hemisphere.

To date, surgical resection remains the main treatment for GB, despite the poor prognosis. Even with the addition of radiotherapy and chemotherapy, 70–80% of patients die of GB within 3 to 6 months of being diagnosed, with a 1-year survival rate of only 10% [1].

The current challenges in the treatment of GB involve difficulties in successfully resecting the tumor [2]. GB is known to be a highly heterogenous tumor, leading to drug resistance [3]. Several systemically administered drugs depict less bioavailability in the central nervous system (CNS), mainly due to the blood–brain barrier (BBB). Therefore, patients with GB require more effective treatments or drug delivery/targeting methods that can overcome the limitations of the current standard care and surpass the BBB [4]. Various cell types display intrinsic tropism to tumor tissue which renders them appealing candidates for targeted anticancer drug/gene delivery. The realm of the genetic modification of cells to release/express anticancer molecules has opened new avenues in GB management.

## 2. Cell Therapy for Glioblastoma

### 2.1. Immune Cell Therapy

A large body of literature provides evidence for the promising potential of immunotherapy in the treatment of GB. Being notorious for its extensive local invasion into deeper areas of the CNS, GB is always difficult to resect. Due to this obstacle, immunotherapy is taking the forefront as a promising treatment option for those diagnosed with GB. Various immune cell types are reported to specifically attack the tumor cells using myriad mechanisms of recognition and destruction. Many of those cells will be discussed separately below.

#### 2.1.1. Lymphocytes/CAR-T

When considering immune cells, it is always important to discuss the role of T and B lymphocytes. Both cell types work together to formulate the immune responses our bodies have towards invasive antigens and substances. They can easily cross the BBB under certain physiological and pathological conditions [5], and they perform well when combating tumors. Nair and colleagues induced certain T cells in vitro to become CMV pp65-specific, thus acquiring the capability to recognize and kill tumor cells at an increased rate [6]. T cells can elicit a powerful mechanism to eliminate internal and external pathogens. Therefore, they are being used therapeutically to manage different malignancies with promising outcomes [5].

In a recent study, Lee-Chang et al. [7] developed a vaccine utilizing B cells activated with CD40 agonism and IFNy stimulation. This vaccine aims to travel to secondary lymphoid organs and increase antigen cross-presentation. As a result, this vaccine promotes the survival and functionality of CD8+ T cells [7]. It was found that when this vaccine was combined with other treatments such as radiation and PD-L1 blockade, this combination was able to elicit immunological memory that prevented the growth of new tumor cells.

The chimeric antigen receptors (CAR-) T cells are a product of genetic engineering in an effort to achieve a long-term outcome in cases of malignancies such as GB. CAR-T cells are genetically programmed to attack tumor cells by recognizing the surface proteins expressed [8]. They are developed to target tumor-associated antigens (TAA), such as interleukin 13 receptor α 2 (IL13Rα2), epidermal growth factor receptor variant III (EGFRvIII), human epidermal growth factor receptor 2 (HER2), and erythropoietin-producing hepatocellular carcinoma A2 (EphA2) [9,10]. These artificial proteins are composed of an extracellular antigen-binding domain, a transmembrane domain, and an intracellular T-Cell signaling domain like Cd3 ζ (with or without costimulatory components) [11]. Different generations of CAR-T cells have been studied with outstanding outcomes in redirecting the cytotoxic nature of T lymphocytes to become independent of the major histocompatibility complex (MHC) restrictions and without requiring antigen priming [12]. First generation CAR-T cells have an antigen recognition domain (scFv) and the activating signal CD3 ζ, a costimulatory molecule, can be added to form a second and third generation CAR with two costimulatory molecules [13]. Recently, Bielamowicz et al. Bielamowicz, Fousek [14] stated that GB cells overexpress different and targetable surface antigens, such as HER2, IL13Rα2, EphA2 and EGFRvIII which have been targeted using CAR-T cells with promising outcomes.

EGFRvIII is a variant of EGFR present in 31–64% of patients with GB that promote tumor cell proliferation, invasion, and angiogenesis in the tumor environment [15]. EFRON vIII CAR-T cells localized to intracerebral tumors reduce the expression of EGFRvIII in cancer cells [16]. Chen et al. [15] generated an EGFRvIII-targeting CAR (806-28Z CAR) using the epitope of 806 antibody, which is only fully exposed on EGFRvIII or activated EGFR, for in vitro and in vivo testing with an SQ xenograft established by injecting 1 × 10^7^ GL261/EGFRvIII cells mixed with Matrigel (4:1) into the right limb of C57BL/6 mice. They reported dose-dependent cytotoxicity against mouse GL261/EGFRvIII cells, effective inhibition of tumor growth, effective lysis of mixed heterogenous GL261 cells accompanied with high concentrations of granzyme B which can be used as a predictive marker to determine the effectiveness of CAR-T cells as a therapeutic approach [15]. Likewise, Ravanpay et al. [17] used EFGR806-CAR through intracranial administration to treat xenograft GB mouse models. Placing the CAR-T cells near the target site lowered the risk of side effects outside the CNS and ensured consistent regression of orthotopic glioma.

HER2 antigen is over expressed in about 80% of GB cases and was incorporated in the design of a third generation anti-HER2 CAR (anti-HER2 scFv-CD28-CD137-CD3ζ) combined with a PD1 blockade and anti-HER2 scFv from the 4D5 antibody for CAR construction to avoid a decreased binding of CAR to antigen [13]. The costimulatory molecule, CD28, induced and increased production of IL-2, enhancing the clonal expansion and endurance of CAR T cells that, when combined with 4-1BB/CD137, were more efficient in INF-γ production and lysis of tumor cells [13]. TanCAR combined two antigen recognition domains for HER2 and IL13Rα2 previously proved to provide a “near-complete tumor elimination” in previous work by Hedge et al. [18]. Higher density of TanCAR-mediated HER2-IL13Rα2 heterodimers was observed on STED super resolution microscopes and confirmed by PLA in addition to increased IFN-γ and IL-2 secretion; all supporting the antitumor characteristic of this therapy [19]. Bielamowicz et al. [14] demonstrated that nearly 100% of tumor cells were killed, in vitro and in vivo, with UCAR-T, a trivalent transgene combining IL13Rα2 binding IL-13 mutein, HER2-specific single chain variable fragment 9scFv), FRP5, and Epha2-specific scFv 4H5 with CD28 as a costimulatory molecule and ζ-signaling domain of the T cell receptor (TCR). The UCAR T cell showed better signaling, increased engagement of a larger domain of GB cells and an almost entire activation and proliferation of the 3 CARs as demonstrated in surface staining [14].

In efforts to evidence long term immunity after therapy with CAR T cells, Pituch et al. [10] proposed the utilization of IL13Rα2-CARCD28ζ CAR-T cells and observed an increased number of CD8α+DC cells known to efficiently cross-present both cell-bound and soluble antigens in the MHC class I, therefore inducing a CD8+ T cell response. Krenciute et al. [9] designed the IL13Rα2-CAR.IL15T cell by modifying T cells with a retroviral vector that encoded an IL13Rα2-specific scFv with CD28.ζ endodomain and a retroviral vector that encoded inducible caspase-9, NGFR with a shortened cytoplasmic domain and IL15 separated by 2A sequence demonstrating that the action of IL15 in IL13Rα2-CAR T cells enhanced their effector functions. Although IL15 did not show any significant improvement in the proliferation of IL13Rα2-CAR T cells or cytokine production after the first antigen-specific stimulation, it showed significant proliferation after the third stimulation [9]. In light of the fact that steroids (e.g., dexamethasone) form part of the standard protocol of treatment for patients with different malignancies for alleviation of symptoms, it was demonstrated that the antitumor response and presence of intracranial IL13BBζ in T cell-treated mice were not significantly impaired after dexamethasone was given, when compared to the control group, by Brown, Aguilar [20].

Another molecule believed to be effective if used with CAR T cells is B7-H3, a type I transmembrane protein encoded by chromosome 15, which has costimulatory and co-inhibitory functions on T cell subsets [21]. B7-H3.CAR with CD28 costimulation showed a faster antitumor effect in comparison with 4-1BB co-stimulation with no markable difference in antiproliferative activity in general [22]. Although both costimulations showed cross-reactivity to murine B7-H3 without toxicity when infused systematically, and antitumor activity, using in vitro and xenograft GB murine models, allowed the elimination of both differentiated tumor cells and cancer stem cells (CSCs). The low expression of B7-H3 in one third of GB cells demonstrated effective killing by B7-H3.CAR-T cells [22].

In an effort to understand the dynamics of the cytotoxic effects of T-cells in GB to establish better delivery of therapies, Murty, Haile [23] used intravital microscopy to evidence the use of CAR-T cells along with focal radiation, achieving complete tumor regression in vivo. In another study, the IV administration of IL13BBζ was shown to be ineffective, possibly due to deficient cell trafficking to the intracranial tumors, pointing out intracranial therapy with CAR-T cells as a better option for long-term survival [20].

Nevertheless, tumor recurrence is the top burden in the development of effective therapies for GB. Patients treated with IL13Rα2-targeted CAR-T cells showed recurrence with loss and/reduced expression of IL13Rα2 reducing the efficacy of the therapy and making it even more difficult after treatments [12]. Moreover, to achieve complete eradication of GB, there are some barriers yet to concur, such as the suboptimal penetration of CAR-T cells within the tumor stroma, the poor effector function of T cells which inhibits a continuous antigen-driven stimulation and the minimal antigen specificity as a consequence of the heterogeneity of the GB tumor that could cause off-site toxicities [23]. There is also a need for further investigation on how to enhance the endurance of CAR-T cells within the tumor environment to eradicate large tumors or even better early detection and eradication of tumor recurrence [22].

#### 2.1.2. Natural Killer (NK) Cells

The use of NK cells is the most preferred immunotherapy approach discussed in the literature regarding the GB treatment. The NK cells can be used for the targeted killing of glioma cells. Further, they can be used in combination with other immunotherapies including inhibitors for immune checkpoints, drugs targeting immune-related genes, or specific antibodies that block the action of proteins protecting NK cells from immunosuppression [24,25,26,27]. Although a large body of evidence suggests a positive effect of NK cells as immunotherapy in GB treatment, the major hurdle is to mitigate the suppression of the cytotoxic effects of NK cells.

A study by Lee et al. reported the potential use of NK cells in the inhibition of systemic metastasis of GB cells in the mice model. This was attributed to the cytotoxic effects of NK cells against GB cells. Therefore, adequate supplementation of NK cells to the brain can be considered as a promising immunotherapy to treat GB [25]. The killer Ig-like receptor (KIR) genotypes in NK cells are correlated with various tumor types. The presence of KIR2DS2 immuno-genotype NK cells was shown to be associated with more potent cytotoxic activity against GB [24].

Another approach discussed in the literature includes the adoptive transfer of CAR-modified immune cells for the treatment of GB. A Han et al. study elucidated the use of CAR-engineered NK cells in the treatment of GB via targeting wild-type EGFR as well as mutant form EGFRvIII. These EGFR-CAR engineered NK cells demonstrated increased tumor cell lysis capacity, stimulated production of IFN-γ, and further suppressed the tumor growth and subsequently improved survival outcome for a long period [28]. The observations of another study published in 2016 were concordant with the above-mentioned evidence [29].

A study by Tanaka et al. reported another approach using a combination of ex vivo-expanded highly purified natural killer cells (genuine induced NK cells (GiNK)) and the chemotherapeutic agent temozolomide for the treatment of GB. This therapy has been shown to help to stimulate anticancer effects including the stimulation of tumor cell death in human GB cells in vitro [30]. In addition, another study demonstrated that the use of the pretreatment approach of GB with another anticancer agent, bortezomib, helped to stimulate the cytotoxicity of NK cells by inducing TRAIL-R2 expression and enhanced GB lysis due to increased IFN-γ release [31]. Recent research involving the treatment of NK cells with IL-2/HSP70 stimulated BBB crossing and the subsequent antitumor effects of NK cells and resulted in a substantial tumor growth inhibition and prolonged survival in an in vivo study [32].

In a study published in 2005, tumor-derived RNA transfected dendritic cells (DCs) were shown to increase the cytotoxic ability of NK-like T cells by recognizing and killing the tumor cells using adaptive as well as innate immune systems, thereby enhancing antitumor effects against the tumor from which RNA was originated [33].

Furthermore, NK cells were used as a vehicle for oncolytic enterovirus delivery in several recently published studies [34,35]. Recently published evidence by Shaim et al. reported an innovative mechanism of NK cell immune evasion by GB stem cells by targeting the integrin-TGF-β axis, leading to its inhibition and consequently improving the antitumor effects of NK cells against GB [36].

#### 2.1.3. Dendritic Cells (DCs)

Many different experiments have utilized dendritic cells (DCs) in different ways to aid in therapies. In a study conducted in 2018, it was found that if DCs were used to mediate the delivery of nano-DOX, it would result in the stimulation of GB cell immunogenicity and this method would result in an antitumor immune response in GB [37]. Additionally, there have also been vaccines created that have improved the survival rates and tumor regression rates by elevating the antitumor immune function. One of these vaccines, named STEDNVANT, was established in 2018 and it was found to upregulate PD-1 and its ligand on PD-L1 effector T cells, DCs, and GB tissues [38]. This resulted in an increase of regulatory T cells in the brain tissue and lymph nodes. When this vaccine was combined with the antibodies of anti-PDL1 it was proven to show a greater survival rate and a decrease in the T-regulatory cell population in the brain [38].

Another study by Peeters and colleagues has found that a specific DC vaccination against H3.3G34R GB did stimulate and increase the adaptive immunity driven by neoantigen specific cytotoxic T lymphocytes. This resulted in increased survival for the test animals [39]. Additionally, Shao et al. [40] have recently found that DCs that were transduced with glioma-expressed antigen 2, via the recombinant adenovirus, had induced cytotoxic lymphocyte response and decreased the tumor growth in mice. Similarly, it was found that DCs, transfected with modified CD133 mRNA, were able to maintain CD8^+^ T cells for a statistically significant period. The DC-based vaccination prolonged the survival of tumor-bearing mice [41].

#### 2.1.4. Monocytes/Macrophages

Another immune cell subtype, monocytes, was also studied and proven to be beneficial against gliomas. In 2017, Wang et al. found that monocytes loaded with nano-doxorubicin were able to successfully cross an artificial endothelial barrier and were able to release drugs in GB spheroids once they got inside [42]. This drug release method was proven to improve the effectiveness of the drugs they were meant to deliver to the mice in this experiment and improved their tumors [42]. Utilizing a similar method, another study was able to deliver monocytes that were loaded with conjugated polymer nanoparticles into GB cells and that allowed for the expansion and improvement of photodynamic therapy for GB [43]. Additionally, another experiment by Gattas and colleagues, [44] used primary monocytes that were cultured in the presence of U87MG-conditioned media, and co-cultured with GB spheroids. They found that monocytes differentiated and acquired clear M2 phenotypes, while also inducing alterations in the cell cultures. The fact that these monocytes upregulated CD206, CD163 and MERTK surface markers on the CD11b and CD14 populations made them strong inducers of anti-inflammatory macrophages [44]. These three studies can be referenced together to show the range of ways that immune cells can be used to provide care to those with life-devastating tumors.

#### 2.1.5. Neutrophils

**Figure 1:**
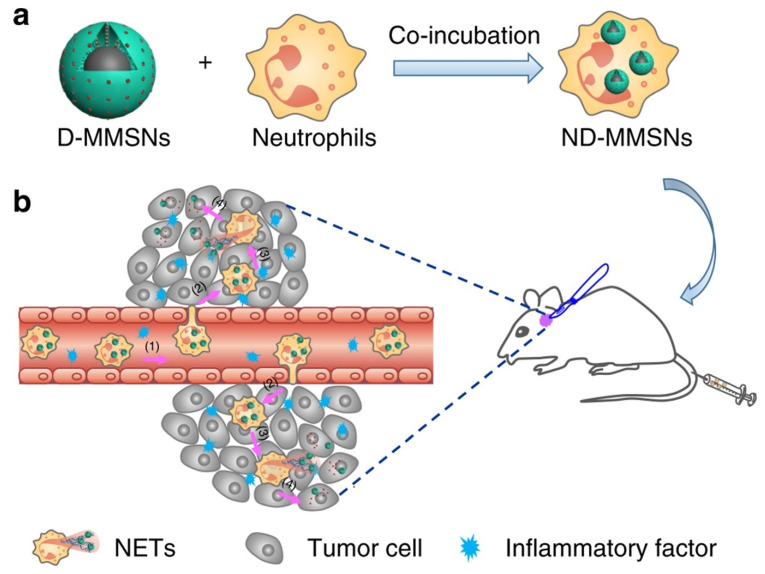
Fabrication and targeted-therapeutic schematics of ND-MMSNs to achieve residual tumor theranostics. (**a**) Schematic illustration of the preparation of ND-MMSNs. (**b**) Schematic shows that inflammation-activatable ND-MMSNs target inflamed glioma sites and phagocytized D-MMSNs would be released to achieve residual tumor theranostics [47] (CC by 4.0).

Neutrophils are considered one of the promising immune system cells that are useful in the intracranial treatment of GB in terms of an effective drug delivery system to the target area by enhanced penetration through the BBB. A recently developed novel approach for GB includes an ultrasound augmented chemo/immunotherapy using a neutrophil-delivered nanosensitizer [45,46,47]. In a Li Y, et al. study, authors introduced a novel design wherein a hollow titania (TiO_2_)-covered persistent luminescent nanosensitizer was used for optical imaging-guided, ultrasound-augmented chemo/immunotherapy against GB. Specifically, neutrophils were used as drug delivery vehicles wherein they were loaded with hollow titania (ZGO@TiO2@ALP-NEs) which can cross BBB and reach target GB cells effectively. The postactivation of this system using ultrasound radiation results in ROS generation from ZGO@TiO2@ALP further causing liposome destruction and drug release at the GB sites. This ultimately leads to local inflammation thereby enhancing the migration of more drug-loaded NEs into the tumor sites for augmented and sustained therapy. This treatment approach showed improved survival in a mice model with GB which provides the benefit of immuno-surveillance of recurrent tumors for a long period [45]. In addition, other studies also provide evidence in support of neutrophils as a promising drug delivery system in GB therapy, as seen in Figure 1 [47].

Despite the great promise immunotherapy has in cases of GB, the disease is quite notorious for its high recurrence rate [48]. The recurring tumor cells depict high heterogeneity and serious radiotherapy/chemotherapy-induced genotoxicity. Moreover, most of the time, those tumors often engage in antigen escape after immunotherapy, thus it is not feasible to apply immunotherapy on recurring GB [49]. For instance, CAR-T cell and vaccination therapies did not achieve satisfactory results in clinical trials on cases with recurrent GB [50].

### 2.2. Stem Cell Therapy

The potential utility of stem cells as sources for cell-based therapy has been viewed as the next generation treatment for GB. The therapeutic effect of various types of stem cells were tested on GB in preclinical and clinical settings. Stem cells are capable of self-replication, differentiation, tumor tropism, and many other features rendering them appealing therapeutic candidates. In addition to their ability to regenerate CNS cells after tissue injury following surgery and/or chemotherapy, they have been believed to exhibit direct/indirect antitumor effects. The preclinical evidence for such therapeutic effects of stem cells in GB is discussed next.

#### 2.2.1. Mesenchymal Stem Cells (MSCs)

In 1966, the existence of mesenchymal stem cells (MSCs) in bone marrow was reported by Friedenstein et al. [51]. The term “mesenchymal stem cells” was proposed by Caplan, in 1991 [52]. This term reflected their ability to differentiate into osteoblasts, chondrocytes, and adipocytes. Later, MSCs were found to be present in many other tissues, like adipose tissue, umbilical cord, menstrual blood, dental pulp, etc. [53,54,55,56].

Overtime, MSCs were recognized to have the inherent ability of self-renewal. However, there was no definite marker for MSCs so far [57,58,59,60]. Therefore, the International Society for Cellular Therapy (ISCT) established the minimal criteria for MSCs that were defined based on their biological features. Firstly, MSCs must have plastic adherent growth. Secondly, MSCs must have positively expressed CD73, CD90, and CD105 surface antigens and have negative expression of CD14 or CD11b, CD 19 or CD 79α, CD34, CD45, and HLA-DR surface molecules. Thirdly, MSCs must show differentiation ability towards osteoblasts, adipocytes, and chondrocytes in vitro [61,62].

Of note, the MSCs in human GB can demonstrate ISCT criteria [63]. However, they can express slightly different cell surface markers. These are desmin, vascular endothelial (VE)-cadherin, α-smooth muscle actin (α-SMA), and nerval/glial antigen [64,65]. This shows that cell surface expression is not limited to ISCT criteria. MSCs taken from different sources have also been shown to differentiate into multiple cell lines under specific in vivo and in vitro conditions [65]. The biological features of MSCs are complex and manifold in their regulation. The main reason for the slight differences in the biological characteristics of MSCs is due to the microenvironment conditions in the different sources. miRNAs are relatively newer mechanisms that can regulate the biological features of MSCs. The microenvironment and signaling pathway interactions of MSCs can be better understood in their role in modulating the biological features of MSCs in the treatment of GB [66].

##### Antitumor Effect of MSCs

Thanks to their appealing features, including their tropism toward malignant gliomas, MSCs are considered a potential vehicle for the delivery of therapeutic agents to GB. The chemoattractant molecules secreted by tumor cells, corresponding receptors, and membrane protein in MSCs govern tropism. These tumor-associated MSCs (TA-MSCs) are recruited into the tumor microenvironment of gliomas to promote tumor growth via cytokines, chemokines, and growth factors for cross-talking among tumor cells [67,68]. Endothelial cell growth factor (EGF), platelet-derived growth factor (PDGF), VEGF, TGF-β1, interleukin (IL)-8, SDF-1α and monocyte chemoattractant protein-1 (MCP-1) are found to be secreted by stromal cells or glioma cells, contributing to the tumor-tropic effects of MSCs [69,70,71]. As a result of the tropism of MSCs, MSCs have been used as a trojan horse as carrying vectors for GB therapy. MSCs have been used by many studies as cell vectors. They deliver antitumor proteins, immune factors, long non-coding RNAs (lncRNAs) or antitumor microRNAs (miRNAs), oncolytic viruses or suicide genes.

##### Cytokine-Based Therapy

This is done by delivering gene-modified MSCs to overexpress cytokines. MSCs can be transduced with nucleic acids coding for various cytokines, for example, interleukins (2, 7, 12, 18, 22, and 24), BMP4, and IFN-β, alone or in combination. At the glioma site there is C4 and CD8 lymphocytic infiltration. This stimulates the antitumor effects by boosting T-cell infiltration [72,73,74,75,76,77,78,79,80,81,82,83,84,85].

##### Oncolytic Virus Therapy

This is accomplished by inserting genetically modified viruses into MSCs. The MSCs then home into the tumor site, the virus is deployed and selectively infects and kills tumor cells [86,87,88,89,90,91,92].

##### Anti-Angiogenesis Therapy

It is known that the inhibition of angiogenesis curbs the growth of tumors. MSCs have been used to express pigment epithelial-derived factor (PEDF). PEDF activates the Fas/FasL pathway resulting in the induction of endothelial cell death. In this way, the regulation of the balance of inhibitors and inducers of angiogenesis can occur. MSCs expressing PEDF can also induce tumor cell apoptosis and inhibit angiogenesis, eventually reducing tumor volume [93,94].

##### Induction of Tumor Cell Death

The MSC-derived secretome can also exhibit cell cycle arrest in GB cells [95]. Moreover, cytotoxic cell death can be achieved by MSCs with expressed tumor necrosis factor (TNF)-related apoptosis-inducing ligand (TRAIL). This causes the activation TNF/CD95L axis which results in the induction of apoptosis of GB cells [55,84,96,97,98,99,100,101].

Induction of cell death can also be achieved by transducing MSCs with mRNA. This mRNA is used to encode a pro-drug-activating enzyme, which functions as a suicide protein. Tumor regression occurs when MSCs laden with the suicide protein are injected into the tumor site. The suicide protein converts the non-toxic pro-drugs into toxic pro-drugs in the tumor site [76,102,103,104,105,106]. Drugs can also have a synergistic effect in inducing cell death [107]. Phosphatase and tensin homolog (PTEN) mRNA-engineered MSCs have also been seen to demonstrate cytotoxic effects [108].

MicroRNAs can also be used to induce apoptosis or senescence when delivered by mesenchymal stem cells [109,110,111,112,113,114,115].

##### Protumor Effects of MSCs

While MSCs show huge promise as an antitumor treatment for GB, they can also promote tumorigenesis. The modulation, migration and invasion of tumor cells have been reported under the effect of BM-MSC-conditioned media. Such conditioned media revealed the expression of six types of proteins (Oat, Calr, Ddah1, Npm1, Set, and Tardbp) in the presence of C6 cells. These are closely related in causing cell differentiation and proliferation. Additionally, nine other proteins (Anxa4, Actr1b, Actn4, Pdia6, Rap2c, Sphk1, Vim, Tuba1c, and Tpm2) associated with the cytoskeleton and motility were expressed [76]. MSCs that infiltrate the GB tumor were probably responsible for tumor growth by releasing exosomes. The tumor growth may be controlled by the reciprocity of tumor cells and stromal cells. Chemotaxis of SDF-1/CXCL12 and MCP-1/CCL2, along with exosomes derived from the MSCs, is believed to govern the process [116]. Similarly, Pavon and colleagues have attributed the tropism and pro-oncogenic effect of MSCs towards CD133^+^ GB cells to the same chemotactic factors SDF-1/CXCL12 and MCP-1/CCL2, along with exosomes derived from the MSCs. Promigratory chemokines from MSCs could aid in tumor growth through angiogenesis, proliferation, epithelial-mesenchymal transition, senescence, immune evasion, and metastasis [116].

Another mechanism proposed by Sun et al. [117] was the fusion of MSC with the GB tumor cells. The fused cells could promote neovascularization in the tumor [117]. Moreover, MSC elicited an increased proliferative and invasive tumor cell behavior under 3D conditions, mostly due toTGFB1 or exosomal proteins from MSCs [118]. In a similar approach, exosomes released from glioma-associated MSCs enhanced the clonogenicity and proliferation of GSC. These exosomes were identified in the intercellular transfer of miRNA leading to downregulation of the tumor-suppressive nuclear receptor corepressor NCOR1 which eventually facilitated the GB aggressive growth [119]. In a recent study by Iser and colleagues [120], when ADSCs were co-injected with C6 cells into the brains of rats, they promoted tumor growth and reduced the rats’ survival in comparison to rats that received ADSC-CM pretreated C6 cells or C6 cells only. This was attributed to the immunomodulatory and immunosuppressive effects of ADSCs encouraging tumor growth. More seriously, the MSCs grown in indirect co-cultures with C6 glioma cells revealed characteristics of tumor transformation. The MSC invasion and migration through STAT3 signaling were promoted by interleukin-22 [78].

**Figure 2:**
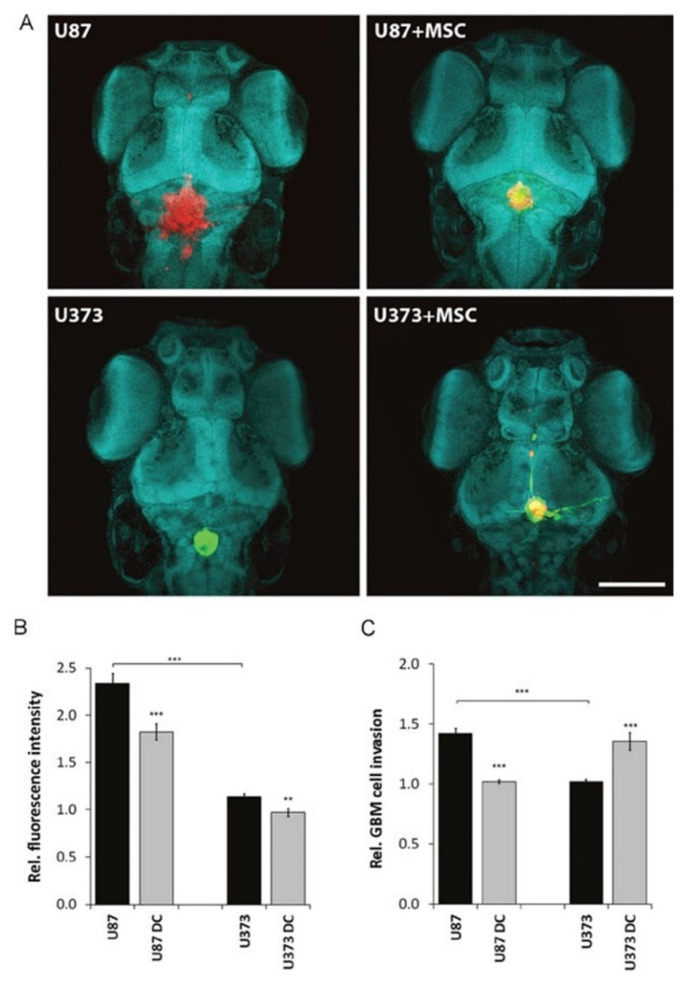
Proliferation and invasion of U87 dsRED and U373 eGFP cells in the zebrafish embryo brain upon co-injection with DiO/DiI-labelled MSCs. (**A**) Two days after zebrafish embryo fertilization, U87 and U373 cells alone (left upper and lower panels) or mixed with fluorescently stained MSCs with DiO (green) in the case of U87 (right upper panel), and with DiI (red) in the case of U373 (right lower panel), were injected into the brains of the zebrafish embryos. Cell nuclei were stained with methyl green (magnification, 10×, green blue shapes; scale bar = 250 μm). (**B**) GBM cell proliferation was determined 24 and 72 h after the injections by confocal microscopy and quantified as relative fluorescence intensity of U373eGFP and U87dsRed labelled cells injected alone or with MSCs (DC). (**C**) Relative invasion of U87dsRed and U373eGFP cells injected alone, or with fluorescently stained MSCs (DC) was determined as described in Material and Methods, clearly showing increased U373eGFP invasiveness and reduced U87 invasiveness from co-culture xenografts. Thirty zebrafish embryos were used per group. Data are means ± SD. ** *p* < 0.01, *** *p* < 0.001 [121]. (CC BY 3.0).

Nevertheless, such tumorigenic effect of MSCs was found to be cell-line dependent. In an interesting study by Barbara Breznik and her team, they found that MSCs inhibited the invasion of U87 cells while enhancing that of U373 GB cell lines (Figure 2) [121].

#### 2.2.2. Neural Stem Cells (NSCs)

Neural stem cells (NSCs) have two main characteristics: the capacity of self-renewal and their potential to differentiate into neural progenitor cells (limited potential, limited self-renewal) including the cells of the neuronal lineage, such as neurons, astrocytes, and oligodendrocytes [122]. Progenitor cells are typically found in the brain and spinal cord and easily differentiate into neural or glial progenitor cells [122]. Alongside the developing brain, the subventricular zone of the lateral ventricle has been identified as a source of adult animal NSC generation and is also subject to biopsy and cell culture [123].

Due to the link between NSCs and GB, NSCs are presently being explored as optimal models to further explore the knowledge of GB. Additionally, the switch seen between neurogenic to gliogenic is characterized by a burst of oncogenic alterations, which has further indicated that transcription factor AP-1—typically seen in basal gene expression—would ultimately inhibit gliomagenesis if it were transiently inhibited [124]. Ultimately, the use of NSCs to explore the progression of glioma tumorigenesis has uncovered imperative information that would be beneficial to the treatment of GB. Additionally, the NSCs have a unique GB tumor-homing property that would make them beneficial for targeted therapies, such as delivering apoptosis-inducing ligands that can be targeted to the tumorigenic cells and work to induce apoptosis on the tumor cells [125,126].

Induced neural stem cells (iNSCs), cells that undergo transdifferentiation from the patients’ skin, are used as drug carriers with an innate tropism to neural cells including GB [127]. These cell derivatives can be genetically engineered to express cytotoxic proteins, which aid in cancer destruction as the cells will naturally migrate towards the cancer-invading area [127]. The iNSCs could carry therapeutic agents such as TNF α, and thymidine kinase [127]. Of these agents, the TNF α is a TNF α-related apoptosis-inducing ligand (TRAIL) that can diffuse to nearby cells [127]. TRAIL, once in the diffused cells, can induce caspase-mediated apoptosis via engagement of the death receptor with negligible off-target toxicities [127]. The other agent, thymidine kinase TK, is an agent that remains inactive until the prodrug valganciclovir is co-administered and is hydrolyzed to ganciclovir [127]. The TK agent of the iNSCs will phosphorylate the circulating ganciclovir into cytotoxic ganciclovir triphosphate and accentuate the method of action of the drug, which is the inhibition of DNA polymerase—killing iNSCs as well as tumor cells [127].

Along the lines of the TK agent, there is also the notion of the bystander effect from lentiviral vectors followed with doxycycline or ganciclovir [126]. The bystander effect is the idea that the NSCs would only act as a bystander, only needed to deliver the vector [128]. The majority of the action is done by the vector that is delivered by the bystander, in this case, the bystander is the NSCs, and the vector is the agent targeting the cancerous cells. The results of the study showed that the lentiviral vector was compatible with human clinical use, though the timing of ganciclovir administration should be taken into consideration—weaker effects are seen when ganciclovir is administered a week following the transfer of the mesenchymal cells and vector [126].

Presently, the examination of lymphocyte-directed treatment is under examination as well as the use of bispecific T-cell engagers (BiTEs) on the efficacy of recruiting T cells and the production of proinflammatory cytokines interferon γ and tumor necrosis factor α (TNFα) [129]. Bispecific T-cell engagers consist of two single-chain variable fragments connected by a flexible linker [129]. One of the singe-chain variable fragments is directed to a thymidine-adenosine-adenosine and the other to CD3 epsilon that is expressed on T cells [129]. Bispecific T-cell engagers have a specificity to tumor therapeutic potential when pairing with a recombinant molecule [129]. Studies have shown that neural stem cells that have been modified to produce BiTEs are capable of recruiting T cells as well as the proinflammatory cytokines interferon γ and tumor necrosis factor α (TNFα) [129]. Additionally, it is also seen that there is the effective killing of GB cancer cells with the NSCs modified with BiTEs, specifically the promotion of T-cell killing of IL13Rα2+ tumor cells by engaging the tumor cell antigen with CD3 epsilon T cells that effectively target them [129]. Overall, NSCs can also be applied as a means for an innate reaction.

As the use of NSCs for the delivery of therapeutics has become a highly explored subject, the efficacy of drug usage has been explored with a three-dimensional culture system to confirm the efficacy as well [130]. Through a three-dimensional culture system, NSCs were analyzed based on tumor location for the effect of their migration [130]. It was seen that when NSCs were implanted 2mm lateral from the tumor foci, they were found to colocalize with multiple tumors and preferred to migrate to the tumor foci that were near the site of implantation [130].

It is additionally observed that NSCs can speed up the tumor formation process [131]. Thus, if a treated NSC that is to be used as a vector proves to be defective, it is possible for the NSC that is introduced to further contribute and move the disease process along [131]. It is postulated that NSCs are self-limited and the process in which the cells are used in treatment additionally includes a termination process that also limits the cells from progressing into a cell line [126].

#### 2.2.3. Induced Pluripotent Stem Cells (iPSCs)

Since Shinya Yamanaka created induced pluripotent stem cells (iPSCs) in 2006, the field of stem cell research has been revolutionized [132,133,134]. With the somatic cell reprogramming technology, it is now possible to reprogram virtually any somatic cells to a pluripotent embryonic stem cell-like state by delivery into the somatic cells of a mixture of reprogramming transcription factors [134]. Like embryonic stem cells (ESCs), iPSCs can proliferate infinitely in culture and will differentiate into the three embryonic germ cell layers (ectoderm, mesoderm, and endoderm), thus can develop into all cells of an adult organism, including neural stem cells (NSCs) [134]. Since ESCs are derived from pre-implantation embryos, their embryonic origin raises strong ethical concerns relating to embryo destruction, thus hampering their clinical application. iPSCs avoid these ethical issues thus opening the way for the progression of pluripotent stem cell research clinically [134].

**Figure 3:**
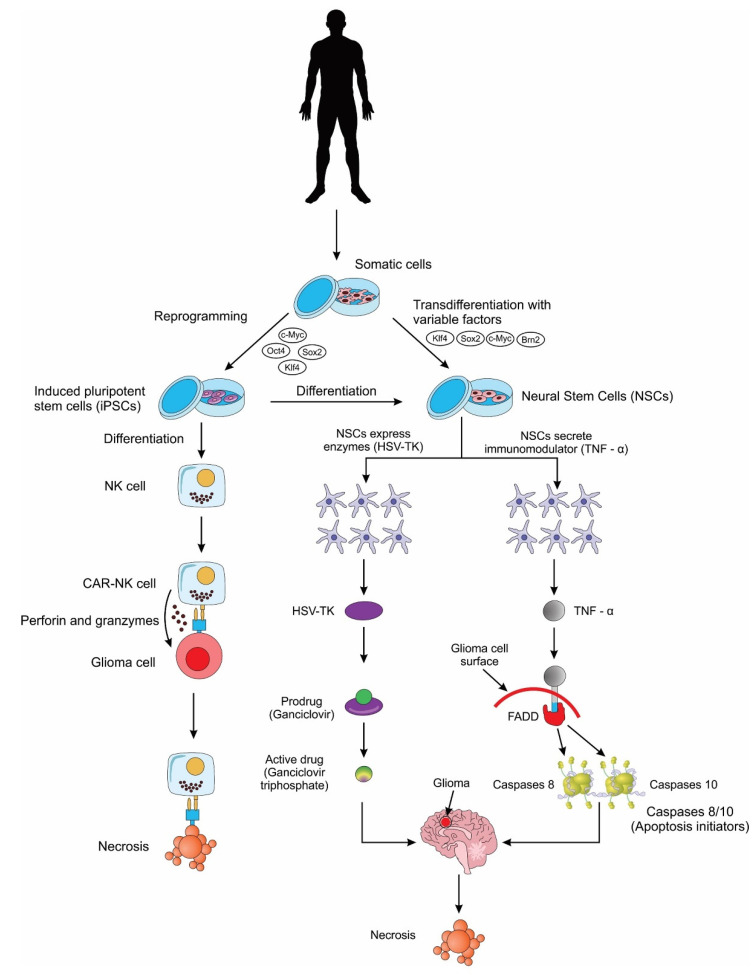
A schematic showing the different applications of induced pluripotent stem cells (iPSCs) and induced pluripotent stem cell-derived neural stem cells (iPSC-NSCs) for the treatment of glioblastoma cells. NK cell: natural killer cell; CAR: chimeric antigen receptor; HSV-TK: herpes simplex virus thymidine kinase; TNF-α: tumor necrosis factor alpha; FADD: Fas-associated proteins with death domain.

Namba and colleagues, in 2014, demonstrated that both iPSCs and iPSC-NSCs had a similar potent tumor tropism following transplantation, thus showing that iPSCs and their derivatives can be useful tools as vehicles of transport in stem cell-based gene therapy for the treatment of GB [135]. In two separate preclinical studies, Bago and colleagues use the process of transdifferentiation (TD) to generate iNSCs for the treatment of glioma [136,137]. TD involves the direct reprogramming of somatic cells into a lineage-specific cell (in this case NSCs), bypassing the dedifferentiation into a pluripotent stem cell. The resulting mouse and human iNSCs were engineered to produce a transmembrane protein called tumor necrosis factor-related apoptosis-inducing ligand (TRAIL). TRAIL can recruit Fas-associated proteins with death domain (FADD), which in turn bind to apoptotic caspases 8/10, thus inducing apoptosis and cell death in malignant cells [138,139,140,141,142,143]. The efficacy of this cytotoxic factor-based therapy study was demonstrated by the decrease in tumor size and the improvement in survival rates of the mice glioma model compared with controls [136,137]. In the second preclinical study, Bago and team tested the widely popular suicide-protein-based therapy method along with the cytotoxic-factor based therapy [137]. The suicide-protein-based therapy, or the enzyme/prodrug strategy involves a gene encoding an enzyme (suicide protein) into the stem cell. Once injected to the tumor site, the enzyme converts the non-toxic pro-drug into a toxic pro-drug which helps to regress the tumor cells. The most used combination is the herpes simplex virus thymidine kinase (HSV-TK) with ganciclovir (GCV). The HSV-TK converts GCV into GCV monophosphate, and this is further phosphorylated to GCV triphosphate. GCV triphosphate is a toxic antimetabolite that inhibits DNA polymerase thus leading to tumor cell death [144]. The HSV-TK/GCV suicide-gene system was further demonstrated recently using human iPSC-derived NSCs and it showed considerable therapeutic potential for the treatment of GB [126]. Interestingly, in another study by Bhere et al. iPSC-derived NSCs were engineered to secrete both TRAIL and HSV-TK and a profound antitumor efficacy was demonstrated [145] (Figure 3).

NK cells are specialized killer cells of the innate immune system, with the natural ability to eliminate abnormal (tumor) cells without prior sensitization (unlike T-cells that require prior sensitization). Scientists have genetically modified human NK cells with CARs to further weaponize them for the treatment of gliomas. A few preclinical studies have now successfully demonstrated the use of iPSC-derived NK cells for the treatment of GB, thus providing proof-of-concept for the use of these cells in future clinical trials [146,147] (Figure 3).

Despite the huge advancements in the iPSC field, there are current limitations and barriers to their clinical translation. There is the risk posed by tumorigenicity, immunogenicity, heterogeneity, and economic issues [148,149,150]. These limitations must be overcome if we are to realize the full potential of iPSC technology. Stem cell researchers have addressed the risk of tumorigenicity through methods such as directed differentiation protocols and purification methods such as positive/negative selection markers using fluorescence-/magnetic-activated cell sorting systems. Immune rejection can be diminished through HLA matching using HLA-homozygous iPSCs lines [151], and the use of universal donor stem cells (HLA cloaking method) [152,153,154,155]. Gene editing technology like CRISPR/Cas9 has been widely used to create isogenic iPSCs, thus addressing the cell line variation problems. While researchers make efforts to address all the current limitations of iPSCs, it would be wise to bank iPSCs of specific and common disorders from patients [134]. The general plan of action should be to generate various allogeneic HLA-homozygous iPSC banks with selected lines that cover most of the world’s population [156,157,158]. Due to the high cost of iPSC production, it is no surprise that most of the organizations involved in iPSC biobanking are mainly funded by the government.

#### 2.2.4. Other Stem Cells

Recent research has found that embryonic stem cell (ESC) exosomes could reprogram human carcinoma cells toward a less malignant cell phenotype. In a 2018 study, Zhu et al. developed a tumor-targeting ESC exosome delivery system by modifying ESC-exos with cRGD peptide for glioma therapy. Their results demonstrated that engineered ESC exosomes possessed excellent GB-targeting ability and significantly decreased the viability of the cancer cells [159]. Another study was performed using ESCs on the premise of manipulating the microenvironment of the surrounding GB. He et al. used ESCs to simulate the microenvironment of embryos and determined that their ability to lessen the degree of malignancy is due to downregulation of the P13K pathway. The study proved that the ESCs’ microenvironment could inhibit the PI3K pathway to promote apoptosis and thus inhibit GB proliferation [160].

Hematopoietic stem cells (HSCs) have also been shown to be a viable therapeutic delivery vehicle. Milkina et al. presented the interaction between HSCs and GB stimulated by TGF-β1 in vitro. TGF-β1 contributes a key role in the epithelial–mesenchymal transition, which allows epithelial cells to metastasize. The results of the study demonstrated the high proliferation rates of GB cells stimulated by TGF-β1 while interacting with HSCs, which indicated the ability of normal CD45+ CD34+ HSCs to regulate proliferation programs [161]. In another study using HSCs, Andreou et al. utilized an MMP promoter in HSC gene therapy to effectively transport TGFβ-blocking peptide to experimental GB in conjunction with irradiation. The data showed that TGFβ-blocking HSC gene therapy combined with IR resulted in a markedly longer survival time in contrast with the control group in vivo [162]. It is well known that adoptive T cell immunotherapy (ACT) is a strong candidate for the therapeutic control of CNS tumors. Wildes et al. illustrated that bone marrow-derived hematopoietic stem and progenitor cells (HSPCs) can differentiate into potent antigen-presenting cells of a DC phenotype and synergistically enhance in situ antitumor immunity [163]. They observed that during ACT, HSPC-derived cells in gliomas depend on T-cell–released IFNγ to differentiate into DCs and activate T cells. Although DC vaccines are proven to induce immune responses in the periphery, the study results illustrated that HSPC transfer generates intratumoral DCs that heighten T-cell responses and promote glioma tumor rejection.

Human bone morphogenetic protein 7 (hBMP7), a part of the transforming growth factor- (TGF-) β superfamily, is known to regulate cell proliferation, differentiation, apoptosis, and antagonize TGF-β signaling [164]. Therefore, it was characterized by its ability to induce the differentiation of brain tumor stem cells. Attia et al. [165] demonstrated human teratocarcinoma NTERA2/D1(NT2) cells transfected with hBMP7 nioplexes are a potential treatment vehicle for GB. The in vitro study exemplified the ability of BMP7-expressing neural precursor cells to lessen the tumorigenicity of glioma cells [165].

## 3. Clinical Trials on Cell-Based Treatment for Glioblastoma

The preclinical data found cells quite attractive candidates for the treatment of GB. Therefore, they have been used in cell-based therapies to prime the immune system, attack GB cells, deliver therapeutic molecules, or exert immunomodulation. To date, a significant number of clinical trials have been carried out, using a wide range of cell types in the treatment of GB. In this review, we have included only those studies that are either ongoing or have already been completed. Immune cell therapy is summarized in Table 1, while stem cell therapy is summarized in Table 2. Information was retrieved from the website http://clinicaltrials.gov (accessed on 23 September 2021) and was last updated on 23 September 2021.

## 4. Future Perspectives

Stem cell- and immune cell-based therapies for GB seem to be promising therapeutic strategies. As mentioned in Section 3, several clinical trials were conducted on patients with GB where stem cells, or genetically modified stem cells were administered into the brain of GB patients at the time of surgical tumor resection. However, preclinical research using stem cells to combat GB has found that the lifespan of stem cells injected directly into the brain parenchyma after surgical excision of the tumor was quite limited, compared to their infusion into healthy brain tissue. [166] Therefore, innovative new strategies are being explored to enhance cell effectiveness or to circumvent their limitations.

Polymeric biomaterial scaffolds have been utilized recently to improve tumoricidal stem cell administration and hence retention in the tumor resection cavity, leading eventually to enhanced therapeutic efficacy against GB. [166] In addition, they had low toxicity and good swelling behavior, yet this usually depends on the chemical moieties of the gel-forming polymers, and the administration routes [167].

Several studies suggested that scaffolds could boost stem cell proliferation in the brain tissue, thus extending their persistence. Among those natural biomaterial scaffolds that have been well studied are collagen, gelatin, [168] and hyaluronic acid [169]. As well, fibrin was studied to enhance the retention of tumoricidal SCs due to its capability to polymerize into a fibrous network [170]. Similarly, the electrospun nanofibrous scaffolds increased the efficacy of stem cell-mediated therapy of surgically resected GB [169,171]. Interestingly, the reported effect was not significantly affected by the scaffold’s degradation rate.

Despite the above-mentioned pros of biomaterials, there are still some roadblocks that need further attention. Such limitations include the non-biocompatibility and non-biodegradability properties of some of the biomaterials explored [172]. Moreover, there may be a rapid burst of drug release during hydrogel swelling and fast drug release from some porous hydrogels [167]. This problem can be overcome by the development of biocompatible and biodegradable hydrogels such as PEG-PLGA-PEGa, or by using polymers that have hydrolysable moieties [173]. The significant risk of the remaining unreacted small-molecule cross-linkers in the formula for hydrogel preparation can be overcome by using a polymer–polymer cross-linking method by the formation of a Schiff base or Michael addition reaction [174].

Nanoparticles (NPs) exhibit a breakthrough diagnostic and therapeutic modality in oncology, as they can be developed to interact with several biological systems with accuracy and specificity. To achieve a targeted distribution at a cellular or intracellular level, nanoparticles can be operational via active targeting which is achieved by multiple techniques. A technique called ligand targeting works by covering the nanoparticle surface with one or multiple ligands, such as transferrin. Guo et al., reported that transferrin-modified polyethylene glycol-poly lactic acid NPs complexed with resveratrol had a promising remedial effect to GB, both in vitro and in vivo, and could be a prospective method for the targeted therapy of GB [175]. In another context, Kefayat and co-workers [176] have reported that folic acid and BSA-decorated gold nanoclusters (FA-AuNCs) can be effective radiosensitizers for intracranial GB radiation therapy. The downside of using NPs alone could be their liability, especially the larger ones, to be engulfed by the phagocytic mononuclear system [177]. Therefore, combining NPs with stem cells seems to be advantageous in enhancing the NP loading capacity, transport through the BBB, and delivery to the hypoxic core of the GB. In their study, Chung et al. reported that ferucarbotran-labeled hMSCs, with their overexpressed EGFR, were captivated by tumorous EGF and more efficiently migrated towards the tumor than the unlabeled cells which lead in effective intrinsic antitumor properties [178]. To date, the role of MSCs, as carriers for NPs, has been investigated [179,180,181,182]. For instance, polymeric NPs (paclitaxel-encapsulated PLGA NPs) were loaded into BM-MSC. The method resulted in improved survival in a rat orthotopic GB model when the NP-loaded MSCs were administered in the contralateral cerebral hemispheres [105]. Related approaches using stem cells in GB management depend on their tumor-homing properties. [183]. Moreover, Suryaprakash et al. [184] tried to integrate the advantages of cell- and NP-based drug delivery, with the tumor-homing properties of MSC to achieve targeted multidrug therapeutics for cancer. They have suggested a hybrid spheroid/nanomedicine system, consisting of spheroids of MSCs entrapping drug-loaded nanocomposites [NC]. Spheroid preparations depicted notable MSC’s tumor tropism and superior loading of multiple types of therapeutic payloads. This system targeted the delivery of both protein and antineoplastic drugs by engineered MSCs and NC. The in vivo migration model demonstrated that the hybrid spheroid had higher NC accumulation in the tumor tissue, compared to the single MSC approach, leading to notable tumor suppression in a heterotopic GB murine model.

Regarding immunotherapy, we herein tried to touch on some of the novel approaches that could be of help in conjunction with the abovementioned cell-based immunotherapies. For instance, Song R. et al. [185] have recently proposed the combination of tumor-targeted PDT and GPX4 blockade (by RSL-3) to elicit ferroptosis of the tumor cells and enhance immunotherapy. They utilized engineered acidity-activatable dynamic NPs for targeted RSL-3 delivery at the tumor site. The proposed approach could be combined with any other cell-based immunotherapy in the treatment of GB. This might combat immune resistance and enhance GB immunotherapy. Similarly, a neoadjuvant administration of inhibitors for the immune checkpoints to another cell-based immunotherapy might open a novel avenue for promoting GB immunotherapy [186]. More recently, the utilization of antibodies targeting Siglec-15 was reported as a novel approach to combat cancer immune escape [187]. Again, further studies will inform us if this tool can be effectively used in combination with another immunotherapeutic. Moreover, the realm of exosome-mediated immunomodulation in cancer is an evolving area of research that might open the way for promoting the traditional cell-based GB immunotherapy [188].

## 5. Conclusions

Although cell therapies for GB have their own potential risks and limitations, they exhibit a more promising potential compared to that of conventional treatment. Despite the plethora of encouraging preclinical studies using immunotherapy for the treatment of GB, there are still many doubts and uncertainties regarding its clinical efficiency, especially with recurrent GB. The GB tumor was found to be more resistant to immunotherapy compared to other tumor types. Such resistance is attributed to myriad factors such as their immune-privileged location within the CNS, low GB immunogenicity, and the immune suppressive local micro-environment. In addition, issues related to the approach, administration regimen, and case selection are still to be resolved. The initiatives for developing effective and innovative treatments for GB are numerous and with variable outcomes. Moreover, future studies are still necessary to add insight into the underlying pathologic features of cell-based immunotherapy-related toxicities. Many new perspectives are still to be probed in search of the optimum individualized GB therapy. A clear understanding is still lacking on how immune-modulating agents could potentially interact or synergize with cell-based treatments. Long-term immunity, off-site toxicity, delivery technique, and immune memory continue to be areas of interest that drive research initiatives.

Likewise, stem cells as a therapeutic tool for GB are faced with myriad challenges. For instance, the correct cell choice among the various types of stem cells with versatile therapeutic effects. Other factors, such as the cell dose, injection route, and administration frequency, remain to be optimized. Last, but not least, the potential tumorigenesis some stem cell types might have requires special attention.

To sum up, efforts to overcome the treatment barriers of tumors with characteristic heterogeneity and a hostile microenvironment, such as GB, are imperative for safe and effective clinical applications.

## Figures and Tables

**Table 1:** Clinical trials on immune cell therapy for GB.

Study ID	Start Date	End Date	Cellular Intervention	Phase	Participants	Cell Type	Cell Dose	Cell Delivery Route	Status
NCT00005813	Mar 1997	Jan 2003	Lymphokine-activated killer cells	I	13	Monocytes/ WBCs	Not specified	IT	Completed
NCT00003185	Aug 1997	Jul 1998	Tumor-draining lymph node lymphocyte therapy	II	40	Lymphocytes	9 × 10^8^ to 1.5 × 10^11^	IV Infusion	Completed
NCT00576537	Mar 2001	Oct 2011	Dendritic cell immunotherapy	II	50	DCs	Not specified	SC	Completed
NCT00068510	Jun 2003	Sept 2012	Therapeutic autologous dendritic cells	I	28	DCs	1, 5, or 10 × 10^6^ DCs	ID	Completed
NCT00107185	Jan 2005	Mar 2010	Biological: therapeutic autologous dendritic cells	I	7	DCs	Not specified	ID	Completed
NCT00639639	Jan 2006	Est. Jan 2022	Therapeutic autologous dendritic cells/ therapeutic autologous lymphocytes	I	42	DCs/Lymphocytes	DCs: 2 × 10^7^ Lymphocytes: 3 × 10^7^	IV/ID	Active
NCT00323115	May 2006	Jul 2013	Dendritic cell vaccine	II	11	DCs	1 × 10^7^ DCs	IN vaccine	Completed
NCT00626483	Apr 2007	Jul 2016	RNA-loaded dendritic cell vaccine	I	34	DCs	2 × 10^7^ DCs	ID	Completed
NCT00576641	May 2007	Apr 2012	Autologous dendritic cells	I	22	DCs	Not specified	SC	Completed
NCT00693095	Sept 2008	Apr 2015	CMV-ALT + CMV-DC	I	23	CMV-ALT ^+/−^ CMV-DCs	3 × 10^7^/Kg CMV-ALT ^+/−^ 2 × 10^7^ CMV-DCs	SC	Completed
NCT00846456	Jan 2009	Feb 2013	Dendritic cell vaccine with mRNA from tumor stem cells	I/II	20	DCs	1 × 10^7^ DCs	ID	Completed
NCT00890032	Sept 2009	Feb 2016	Brain tumor stem cell mRNA-loaded DCs	I	50	DCs	2 × 10^6^, 5 × 10^6^, or 2 × 10^7^	ID	Completed
NCT01006044	Oct 2009	Aug 2014	Autologous DCs	II	26	DCs	Not specified	SC	Completed
NCT01081223	Apr 2010	Mar 2011	Activated white blood cells + cancer vaccine+ immune adjuvant activated WBCs	I/II	14	Activated WBCs	N/A	IV infusion	Completed
NCT01171469	Sept 2010	Jun 2012	DCs	I	8	DCs	5, 10 or 15 × 10^6^ DCs	ID	Completed
NCT01109095	Oct 2010	Mar 2018	HER.CAR CMV-specific CTLs	I	16	T-lymphocytes/ DCs	-CMV-ALT (3 × 10^7^) - CMV-DCs (2 × 10^7^)	SC	Completed
NCT01204684	Oct 2010	Jan 2023	Autologous tumor lysate-pulsed DC vaccination	II	60	DCs	1, 5, or 10 × 10^6^ DCs	ID	Active
NCT01280552	Jan 2011	Dec 2015	Autologous dendritic cells pulsed with immunogenic antigens (ICT-107)	II	124	DCs	Not specified	ID	Completed
NCT01588769	Aug 2011	Apr 2013	ALECSAT cell-based immunotherapy	I	23	NK/ Cytotoxic T-lymphocytes	10 × 10^6^ to 1 billion cytotoxic T cells and NK cells	IV Infusion	Completed
NCT01454596	May 2012	Jan 2019	(EGFRv) III CAR transduced PBL	I/II	18	WBCs	Not specified	IV Infusion	Completed
NCT01808820	Aug 2013	est. Nov 2023	DC vaccine	I	20	DCs	1.2 to 12 × 10^6^ DCs per dose	ID	Active
NCT01957956	Nov 2013	est. Nov 2021	Malignant glioma tumor lysate-pulsed autologous DC vaccine	I	21	DCs	Not specified	ID	Active
NCT02049489	Dec 2013	Mar 2017	Autologous vaccine of DC pulsed with purified peptides from CD133 cancer cells (ICT-12)	I	20	DCs	Not specified	ID	Completed
NCT02010606	Jan 2014	est. Jan 2022	DC vaccination	I	39	DCs	Not specified	SC	Active
NCT02366728	Oct 2015	Aug 2020	Unpulsed DCs, Td, human CMV pp65-LAMP mRNA-pulsed autologous DCs, 111In-labeled DCs	II	100	DCs	-1 × 10^6^ autologous unpulsed DCs -2 × 10^7^ hCMV pp65-LAMP mRNA-pulsed autologous DCs	ID	Active
NCT02529072	Jan 2016	Dec 2019	DCs	I	6	DCs	Not specified	ID	Completed
NCT02799238	Mar 2016	Feb 2020	ALECSAT	II	62	ALE Cells	Not Specified	IV	Completed
NCT02661282	Jun 2016	est. Jun 2021	Autologous cytomegalovirus-specific cytotoxic T-lymphocytes	I/ II	65	T-lymphocytes	Not specified	IV Infusion	Active
NCT02820584	Sept 2016	Jun 2017	GSC-loaded autologous dendritic cells	I	20	GSC-DCs	-1st vaccine: 20 × 10^6^ DCs -2nd and 3rd: 10 × 10^6^ DCs -4–6th vaccine: 5 × 10^6^ DCs	Vaccine	Completed
NCT03400917	Jun 2018	est. Feb 2023	AV-GBM-1	II	55	DCs	Not specified	SC	Active
NCT03615404	Oct 2018	Jul 2020	CMV-DCs with GM-CSF	I	11	CMV-DCs	Not specified	Vaccine	Completed
NCT03726515	Mar 2019	Feb 2021	CART-EGFRvIII T cells	I	7	T-lymphocytes	Not specified	IV Infusion	Completed
NCT03360708	Jun 2019	Dec 2022	Malignant glioma tumor lysate-pulsed autologous DC vaccine	I	20	DCs	Not specified	ID	Active
NCT04489420	Oct 2020	Feb 2024	Cryopreserved, allogeneic, off-the-shelf, NK cells (CYNK001)	I	36	NK cells	For IV 1.2 × 10^9^ cells/dose For IT 2 × 10^8^ +/− 5 × 10^7^ cells	IV Infusion/ IT	Active

**Table 2:** Clinical trials on stem cell therapy for GB.

Study ID	Start Date	End Date	Cellular Intervention	Phase	Participants	Cell Type	Cell Dose	Cell Delivery Route	Status
NCT00002619	Sept1994	Apr 2000	Autologous peripheral blood stem cell transplantation	II	60	PBSCs	Not specified	IV Infusion	Completed
NCT00008008	Sept1997	May 2008	Transplantation of autologous PBSCs	II	40	PBSCs	Not specified	IV Infusion	Completed
NCT00014573	Aug 1998	Oct 2004	Transplantation of PBSCs or BM stem cells	II	30	PBSCs/BMSCs	Not specified	IV Infusion	Completed
NCT00179803	Mar 1998	Sept 2009	Transplantation of autologous PBSCs	II	24	PBSCs	Not specified	IV Infusion	Completed
NCT00003141	Mar 1998	Oct 2011	Transplantation of autologous PBSCs	I	94	PBSCs	Not specified	IV Infusion	Completed
NCT00005796	Feb 2000		Fibronectin-assisted, retroviral-mediated modification of CD34+ peripheral blood cells with O6-methylguanine DNA methyltransferase	I	10	CD34+ PBSCs	Not specified	IV Infusion	Completed
NCT00005952	Aug 2000	Nov 2005	Transplantation of autologous PBSCs	II	30	PBSCs	Not specified	IV Infusion	Completed
NCT00025558	Oct 2000	May 2007	Transplantation of autologous peripheral blood stem cells	I	30	PBSCs	Not specified	IV Infusion	Completed
NCT00078988	Oct 2004	Sept 2006	Transplantation of autologous PBSCs	III	1	PBSCs	Not specified	IV Infusion	Completed
NCT00253487	Aug 2005	Aug 2012	Transplantation of peripheral blood or bone marrow CD34-positive stem cells transduced with the MGMT gene	N/A	12	CD34-positive PBSCs/ CD34-positive BMSCs	5 × 10^6^	IV Infusion	Completed
NCT00669669	Feb 2009	Jan 2021	In vitro-transfected (Phoenix-RD114 pseudotype vector) peripheral blood stem cell transplant	I/II	12	CD34+ HSCs	Not specified	IV Infusion	Completed
NCT01172964	Aug 2010	Feb 2015	E. coli CD-expressing genetically modified neural stem cells	I	15	NSCs	Not specified	Brain injection	Completed
NCT03072134	Apr 2017	Dec 2021	Neural stem cells loaded with an oncolytic adenovirus	I	13	NSCs	First cohort 5 × 10^7^ NSCs, second cohort 1 × 10^8^ NSCs, third cohort 1.5 × 10^6^ NSCs	Injected into the walls of the resection cavity	Active

## Data Availability

Not applicable.

## Abbreviations

ALE	Autologous lymphoid effector cells
ALECSAT	Autologous lymphoid effector cells specific against tumor
AD	Adipose tissue
CAR-T-	Chimeric antigen receptor T cells
BM	Bone marrow
CD	Cluster of differentiation
CMV	Cytomegalovirus
CMV-ALT	CMV-autologous lymphocyte transfer
CNS	Central nervous system
CSCs	Cancer stem cells
CYNK-001	Human placental hematopoietic stem cell-derived natural killer cells
DC	Dendritic cells
EGFRvIII	Epidermal growth factor receptor variant III
GB	Glioblastoma
GPX4	Glutathione peroxidase 4
GSC	Glioma stem cell
HER2	Human epidermal growth factor receptor 2
ID	Intradermal
IL	Interleukin
IN	Intranodal
IT	Intratumoral
IV	Intravenous
MSC	Mesenchymal stem cell
NK cells	Natural killer cells
NSCs	Neural stem cells
PBMCs	Peripheral blood mononuclear cells
PBSCs	Peripheral blood stem cells
PDT	Photodynamic therapy
SC	Subcutaneous
UC	Umbilical cord
UCB	Umbilical cord blood
WBCs	White blood cells
